# “…It just broke me…”: exploring the psychological impact of the COVID-19 pandemic on academics

**DOI:** 10.1186/s40359-022-01008-y

**Published:** 2022-12-05

**Authors:** Lynette Thompson, Cindy Christian

**Affiliations:** School of Humanities and Social Sciences, Faculty of Humanities and Social Sciences, The Independent Institute of Education, Cape Town, South Africa

**Keywords:** Academics, Covid-19, Psychological impact, Pandemic

## Abstract

**Background:**

The declaration of COVID-19 as a global pandemic by the World Health Organisation (WHO) in 2020 catapulted institutions of higher education into an emergency transition from face-to-face to online teaching. Given the nature of the COVID-19 pandemic and the continuing after-effects thereof, the study explored the psychological impact of the COVID-19 pandemic on academics.

**Methods:**

A qualitative phenomenological research design was used to explore the psychological impact of the COVID-19 pandemic on academics. Data were collected by means of semi-structured interviews from a sample of 11 full-time academics permanently employed at six public and private higher education institutions in South Africa in 2020 and 2021. The data were analysed by means of thematic analysis.

**Results:**

The study found that the COVID-19 pandemic and lockdown restrictions had a largely negative psychological impact on academics in higher education. The most dominant negative emotions reported by participants included stress, anxiety, fear and guilt either due to the threat of the virus itself, potential for loss of life, lockdown restrictions, a new working environment, and/or their perceived inability to assist their students. Participants also reported feelings of emotional isolation and an increase in levels of emotional fatigue.

**Conclusion:**

In conclusion, institutions of higher education need to be aware of the negative psychological impact of COVID-19 on academics, and ensure they create and foster environments that promote mental well-being. Institutions may offer psychological services and/or emotional well-being initiatives to their academic staff. They must create spaces and cultures where academics feel comfortable to request and seek well-being opportunities. In addition to mental and emotional well-being initiatives, institutions must provide academics with tangible teaching and learning support as this would go a long way in reducing much of the stress experienced by academics during the pandemic.

## Background

In March 2020, the World Health Organisation (WHO) declared COVID-19 a global pandemic [1]. In response to this declaration, the South African government announced a state of emergency and implemented lockdown measures for most citizens. Institutions of higher education were catapulted into an emergency transition from face-to-face contact teaching to online distance teaching. This change was implemented globally [2]. The lockdown, which meant working from home, and switching to online teaching, left many academics reeling as they tried to navigate their new working environment [3]. Given the nature of the COVID-19 pandemic and the continuing after-effects thereof, it is important to explore and understand the psychological impact of the COVID-19 pandemic on academics in higher education.

Research shows that university academics experienced the pandemic in phases, one of which was uncertainty and instability where they had to adjust to the demands of their new working environment [4]. Many academics experienced a fear of online teaching, increased workloads, and increased demands from students and university management. The study lists how academics experienced these stressors and the associated fatigue as the next phase in their experience of the pandemic [4]. Worry, fear, and fatigue were also experienced in response to other factors that accompanied living in a time of a pandemic. This finding is corroborated by a UK study that reported high levels of worry and fear in the early stages of the pandemic [5]. One of the main fears that academics had to contend with during the pandemic was the unexpected propulsion of academics and students into a new level of engagement with technology [2, 6]. This transition occurred haphazardly but out of necessity. One author postulated that most academic staff at universities offering contact classes typically lacked the necessary experience in the pedagogy of online learning [6]. Many academics also reported anxiety over inadequate technological access and resources for themselves and their students [2]. This challenge was particularly pertinent to the South African context where most of the country’s population live in poverty.

The new work/life environment also brought about many new challenges. During the COVD-19 pandemic, the ability to differentiate between work and home life was demanding for academics. Meeting work deadlines, home-schooling their own children as well as running the household were challenging for many [3]. This compounded anxieties already experienced by the pandemic [7]. In addition to navigating their new work/life environment, studies show that academics were living with constant worry and fear over their health and that of their loved ones [8].

The United Nations has warned that the psychological effects of the pandemic, and lockdown restrictions, are underestimated and people should take care of their mental health and well-being [4]. The situation is even more dire in South Africa where many people have historic trauma and live in poverty, and the added burden of the pandemic exacerbated the risk of mental illness. It is also reported that mental health has been neglected during the pandemic as there has been an understandable focus on the physical well-being of populations [4]. The stress associated with the lack of technological and pedagogical readiness to teach completely in an online space, in conjunction with other stressors linked to the pandemic, led many academics to feel drained and emotionally depleted [7]. However, research shows that psychological impact is influenced by coping mechanisms employed by individuals [9], and that these coping mechanisms could account for decreased levels of worry and fear in the later stages of the pandemic [10].

Given the nature of the COVID-19 pandemic and the continuing after-effects thereof, the researchers explored the psychological impact on academics in higher education.

## Methods

A cross-sectional, exploratory research study was conducted as it allowed the researchers to gather and explore data from multiple participants at a single point in time, based on appropriate inclusion criteria [11]. This allowed the researchers to explore the experiences of academic staff in public and private higher education institutions in South Africa.

This research was conducted through the lens of an interpretivist paradigm. The interpretivist paradigm argues that reality is fluid and not fixed; realities are therefore subjective where people are studied in their natural settings. According to one author [11], qualitative research can be used to better understand participants’ views and make meaning of their experiences, contexts and world. Thus, a qualitative phenomenological research design was used to explore the psychological impact of the COVID-19 pandemic on academics. A phenomenological research design was appropriate for this study as it allowed the researchers to access participants’ lived experiences of the COVID-19 pandemic.

### Participants

The sample for the study was purposively selected based on specified inclusion criteria. The sample comprised 11 full-time academics employed at six different higher education institutions in the Western Cape (South Africa). During the recruitment process, each participant was asked to report their institution of employment. This information was securely stored. Selection criteria was as follows: being permanently employed at a South African higher education institution in 2020 and 2021. Three of the 11 participants were male and eight were female. The sample was drawn from the following academic disciplines: Psychology; IT; Commerce; Optometry and Law. Table 1 below outlines the participants’ demographics, depicting their discipline and job category:

**Table 1:** Participant demographic information

Participant	Discipline	Job category
1	Marketing and Management	Lecturer
2	Psychology	Lecturer
3	Psychology	Lecturer
4	Psychology	Lecturer
5	Finance, risk management and business management.	Academic Management
6	Law	Academic Management
7	Commerce	Academic Management
8	IT	Academic Management
9	Financial accounting and finance and taxation	Lecturer
10	Optometry	Lecturer
11	Financial Accounting	Academic Management

### Instruments

The interviews conducted in this study were guided by a semi-structured interview guide comprising seven questions. The broad domains that were explored during the interviews included: the impact of lockdown restrictions; operational changes to the teaching and learning environment; psychological impact and coping mechanisms. Examples of some of the semi-structured interview questions that participants were asked were as follows: “*What was your initial reaction to hearing about the lockdown restrictions associated with the COVID-19 pandemic?”, “Were there any changes to the teaching and learning environment and assessment strategy at your institution and what was the impact of these changes?” and “What does work-life balance mean to you?” and “How were you impacted by the pandemic emotionally and/or psychologically?”.*

### Procedure

Participants were recruited using LinkedIn, a professional, business-oriented social networking platform used by professionals across various industries. This invitation was posted on both researchers’ personal LinkedIn profiles. An invitation to participate in the study was shared with prospective participants who met the inclusion criteria. In the invitation, participants were asked to complete an online consent form which was securely stored in a password-protected Drive that is only accessible by the co-researchers. Each researcher was assigned a set number of participants to initiate contact to set an appointment to conduct an online interview at a time convenient for the participants. Research interviews were split amongst the two researchers (one conducted six and the other conducted five interviews). The duration of the online interviews ranged between 12 and 45 min. All interviews were transcribed by a professional transcription company and the data were securely stored in the online Google Drive.

### Data analysis

Data were analysed using thematic analysis (TA) [12]. When using TA, researchers try to identify, analyse and report on prominent themes or patterns within the data [12]. Thus, in this study, both the researchers identified and analysed the codes and themes related to the psychological impact of academics at higher education institutions.

This process was conducted manually using a Microsoft Excel spreadsheet to sort and colour-code the data. Undertaking this process manually allowed the researchers to engage in in-depth discussions about the data and ratify the process of assigning and naming of themes. Themes were assigned based on previous literature to ensure alignment in the data. Where new information emerged in the data, the researchers engaged in in-depth discussion to assign the most relevant theme.

### Ethical considerations

Ethics approval to conduct the study was obtained from the Independent Institute of Education (IIE) which is the affiliated organisation of the researchers of this study. Participation in the study was voluntary and written informed consent was obtained from all participants. Participants were asked to complete an online confidentiality agreement which was stored in a password-protected location. All participants were informed about the purpose of the study and their right to withdraw at any point, without any consequences. Participants’ anonymity was upheld throughout the research process and all personal identifiers were removed. Participants were referred for counselling support if needed.

### Legitimation and trustworthiness

In qualitative research, reality is a social construct. Therefore, the goal is rather to achieve a measure of trustworthiness as a form of legitimation of one’s research. To achieve a measure of trustworthiness in this study, the researchers applied Lincoln and Guba’s (1985) criteria [13]. Credibility was achieved in this study through the process of triangulation. Comparative analyses were conducted by both researchers who repeatedly identified patterns in the data. Patterns or themes that emerged from the data such as the impact of the lockdown restrictions or the psychological impact of the COVID-19 pandemic. Legitimacy and trustworthiness were also achieved through the transferability of the findings (patterns) in this study. The researchers conclude that the findings of this study may be transferred to other settings with similar contexts, such as academics in other universities during the time of the COVID-19 pandemic.

The researchers employed a process of reflexivity throughout the entire data collection and analysis process and aimed to ensure that their personal experiences did not impact or bias the findings of the study [14].

## Results

The study explored the psychological impact of COVID 19 on academics working in higher education institutions in South Africa. The following section entails the themes that emerged from the data analysis which include:

### Impact of lockdown restrictions

A few participants shared that they experienced several emotions in relation to the announcement of the lockdown restrictions, which was largely related to the initial 21-day lockdown period. Participants responded as follows:

P4: “…daunting…horror…shock…anxiety”

P6: “Initially a lot of fear and anxiety around the unknown”

Many participants reported increased stress and anxiety. They reported that the stress and anxiety resulted mainly around the fear of the unknown in terms of the pandemic and the lockdown restrictions and worry about their health and that of their loved ones. The misinformation in the media further amplified the fear and anxiety.

P9: “so the anxiety now is coming from all those different sources around where you are, as you speak to your students and you wonder so what’s going to happen to me, given that…not just to say is this pandemic going to end or the loved ones that are passing away, but also economically you know, what’s going to happen. So those are some of the things that fed into the anxiety in my own personal experience."

Many of the participants shared their struggle of trying to implement boundaries in the workspace. The participants expressed their difficulty with switching off from work while working from home. One participant shared the following excerpt:

P3: “I found it really difficult to switch off because now your home is your office…boundaries are very difficult to maintain working from home, so fatigue was the order of the day”.

The participant shared that a consequence of blurring the lines was constant fatigue. This sentiment was shared by many other participants.

Participants also reported quite a lot of guilt as they felt that they were not spending enough quality time with family where they could give their undivided attention. They reported being unable to be emotionally available to their families as a result of the constant exhaustion and fatigue due to work and home responsibilities.

Some participants went as far as to self-diagnose and claimed to be suffering from depression. They did, however, acknowledge that they are self-diagnosed. While others reported that they were emotionally depleted due to the unending pandemic, underpinned by a false sense of hope that things would be better in 2021. They seemed to have held onto the new calendar year as the turning point for the end to the pandemic. When they realised this was not to be the case, the emotional depletion set in, and hope started to diminish. One participant even reported that their empathy for the students diminished due to mental exhaustion. This participant reported that due to their personal state of mental fatigue, their interest and care for the well-being of their students had reduced. Another participant reported that they had a pre-existing mental illness that was exacerbated by the pandemic:

P6: “I do suffer with anxiety and my anxiety was just pushed to the limit during the lockdown”.

Some participants reported that the pandemic and lockdown restrictions did not have any negative impact on their emotional and/or psychological well-being. They reported, however, that they experienced some financial impact albeit it was not always a direct impact - it was more related to extended family members. This, they claimed, did have an emotional impact on them though. Some reported that because they did not lose a loved one or “death was not close to home”, they were not conscious of any emotional and/or psychological impact on their well-being.

Thus, some participants reported that there was no emotional and/or psychological impact on their well-being. They considered themselves to be rather resilient during these adverse times:

P4: “I’m resilient, I can cope, I can…I’m able to deal with new challenges and I’ve grown because of it, ja and the adaptation, that agility for me was like a big boost to my self-confidence”.

For many participants, this is a positive side effect of the trials and tribulations of what they faced during the pandemic.

### Changes to the teaching and learning environment

Most of the participants reported that a major change to the teaching and learning environment at their institution was centred around the move from predominantly face-to-face (i.e. contact) teaching and learning solely online. A few participants mentioned experiencing a sense of emotional isolation due to a lack of support from their institution. They felt unsupported by the institution in terms of the way changes were managed, unrealistic expectations (spoken and unspoken) and a lack of regard for personal wellbeing. This resulted in the participants feeling alone, experiencing panic and a heightened feeling of anxiety related to how best to navigate the implications that the lockdown restrictions had on the teaching and learning environment. The participants reported their experiences of 2020 as follows:

P1: “2020 was a rough year” and P7: “It was a very traumatic year”

This upheaval and disruption impacted lecturers and students in various ways. Both lecturers and students had to navigate technology and learning management systems in ways they did not need to in the days before the pandemic. The adaptation to online teaching left some lecturers feeling ill-prepared and anxious at the daunting task ahead:

P5: “…the switch to online was nerve-wracking”

Participants also reported feeling troubled and burdened when they could not assist their students with technological barriers, especially those that were due to socio-economic reasons, such as lack of connectivity, lack of efficient fibre, lack of devices.

Participants reported an increase in workload that was due to the adaptation to a new unfamiliar teaching and learning mode of delivery, supporting students as well as supporting fellow lecturers. One participant relayed the experience as follows:

P8: “it just broke me…the admin behind it was insane”

The participant explained that the marking load associated with the changes in the assessment strategy was exponential, resulting in increased stress levels. Participants also reported that the ever-changing, reactive nature of institutional decisions was rather stress-inducing and meant that the initial weeks and months of the lockdown were profoundly taxing - emotionally and physically.

P7: “Things changed very fast…. everything was so fluid” and “I even forgot what it feels to relax and what it feels to be at peace, what it feels to be happy…where there’s calmness…”.

One participant reported experiencing feelings of guilt related to all the changes that took place in terms of the lockdown restrictions. This guilt was also linked to the implications thereof for the way the participants had to disperse working hours throughout the day. Additionally, this sense of guilt was related to the different roles that the participant needed to fulfil (i.e., related to work and family). One participant explained that the severity of the stress associated with the day-to-day challenges was further exacerbated by the lockdown restrictions and the constant threat of contracting the coronavirus. This resulted in a negative impact on the participant’s physical and mental health.

### The psychological impact of the pandemic on academics

Participants felt that there was no real demarcation between 2020 and 2021. Participants’ perceived lack of ‘closure’ of 2020 felt like 2021 was merely an extension of 2020. This had real implications for academics’ emotional fatigue and exhaustion levels. Many reported that they were emotionally depleted from the negative impact of the pandemic and ensuing lockdown restrictions.

P6: “I feel like this year sort of rolled on from 2020, again there was no real break, there was no switching off…”.

P8: “The students are tired, and I am tired…because none of us could recover from 2020”.

This emotional fatigue was compounded by the fact that participants felt that in 2021 their institution did not adequately address the challenges and issues that were encountered in 2020; they felt there was a missed opportunity in 2021 to reflect on lessons learned from 2020 and ensure a less traumatic experience for lecturers and students alike. They felt as if there was a sense of institutional denial in what had occurred in 2020. One participant shared the following excerpt:

P1: “our socio-economic culture that we have in South Africa, and obviously we have quite a big divide between students who financially are able to afford internet and laptops and all of that, and students who aren’t.“

This participant expressed concern for students who did not have the financial resources to adapt to the online learning environment. Further to this, the participant also shared a feeling of helplessness related to not knowing whether students were accessing the content and their understanding of the content.

### Coping mechanisms employed by academics

One participant shared that not dealing with the reality of the situation was a means of coping. The following excerpt was shared:

P8: “I adapted to that by not dealing with it”

The participants experienced a sense of avoidance in relation to all the changes which were implemented at their institution. Other participants reported using different coping mechanisms since the start of the pandemic. One participant shared that employing self-reflection practices such as daily positive affirmations was very helpful. The participant explained that it was important to reinforce boundaries with themselves and others, especially given that the physical demarcation of work and home became enmeshed. The participant shared that this meant choosing not to answer work emails after a particular time in order to safeguard quality time with family. The following quote demonstrates this:

P6: "I try to reinforce boundaries with myself”

One participant used faith/spirituality (i.e. prayer) as a coping mechanism while navigating many difficult challenges related to the pandemic. A few participants mentioned that accepting the situation and the realities associated with the pandemic and lockdown restrictions helped them to process what was happening and cope.

Some participants relied on the following coping mechanisms: increased alcohol consumption, listening to music, watching Netflix and reading. For some of these participants, these were new coping mechanisms employed at the start of the pandemic and for others, the coping mechanisms were already used but were used more intently. Many of the participants reported that they implemented physical activity (i.e. yoga and exercise at home) as a coping mechanism. A participant reported that consciously getting sufficient sleep in the evening as a means of coping with the daily responsibilities and changes that occurred at a rapid pace. One participant shared that they tracked the number of COVID-19 infections and deaths on a daily basis as a means of coping. The participant explained that this helped with identifying the ‘enemy’ and understanding the reality of an unfolding situation. Another participant shared that their coping mechanism was undertaking personal studies to escape the reality of the pandemic. The participant explained that studying was very helpful as a means of maintaining a semblance of normalcy. There were also a few participants who did not consciously make use of any coping mechanisms during the lockdown period. There was one participant who shared that they were just focused on surviving till the end of the year (when the pandemic would presumably come to an end). The following quote demonstrates this:

P5: “we just had to make it to the end of the year”

A few participants shared that they found comfort by caring for others and helping their colleagues to navigate all the changes that occurred in their respective institutions. This was a way of coping as participants found that this instilled a sense of hope for them. Others explained that helping others inadvertently helped them find a sense of meaning and purpose. These eudaimonic acts provided these participants with a sense of validation. One participant shared the following experience:

P2: “I think I’m also getting used to it, although it’s not healthy for my side because I can support a lot of students as much as I can but what about me, what am I doing for myself”.

During the interview, the participant identified that helping students and availing themselves to students constantly throughout the period of transition was a means of coping, however, they realised that this was a sacrifice of personal wellbeing in the process.

A few participants shared that connecting with loved ones (i.e. virtually or face to face when allowed) was an important means of coping during the lockdown period. Another participant shared that engaging with therapy throughout the lockdown period was very helpful with managing anxiety associated with the pandemic. In order to cope, a participant shared that planning was helpful during the pandemic because the demarcation between work and home life was compromised by the lockdown restrictions. The following excerpt is evidence of this:

P6: “…it sort of gives me that sense of security knowing that this is what I can control and this is what I’m planning to do for the next”.

The participant went on to explain that planning the things that needed to be done on a daily basis helped them feel a sense of control and a sense of achievement once a task was complete.

On a positive note, however, for some participants, there was a definite acceptance of reality and the new order of things in the pandemic. They reported that they had learned to adapt to the new way of teaching, and they felt more adept in their ability to deliver quality online teaching.

P9: “here’s a marked difference in terms of the systems, routine pattern of doing things, things have sorted of liked settled in…”.

This gave them a sense of achievement as their confidence in being able to get students to engage in the online space grew. This was a real struggle for many participants in 2020 as they linked student disengagement to their identity as an effective lecturer.

## Discussion

This study aimed to explore and better understand the psychological impact of the COVID-19 pandemic on academics. Most participants reported that the COVID-19 pandemic and the associated lockdown restrictions had a significant psychological impact on them. The dominant emotional responses reported by participants included stress and anxiety, fear (of the unknown), worry (related to contracting the coronavirus and the wellbeing of family members), guilt (associated with blurring the lines between work and home life). Most participants shared this sentiment given the physical changes related to where they worked and having to adapt to online learning instantaneously. As seen in this study, the consequences of blurring the lines left some participants feeling guilty for not being emotionally available to those who needed them. This finding is corroborated by previous studies that also found that the participants struggled to demarcate work and home life and in trying to find this balance, many of them ended up feeling stressed [3, 4].

The sudden change to online learning at higher education institutions occurred across the world [2]. The results of this study showed that most of the participants experienced the change as stressful. Having to adapt their teaching and learning strategy, while learning to use technology in a meaningful way significantly contributed to the psychological strain. Additionally, participants shared a deep sense of concern for the wellbeing of their students given the pre-existing challenges (i.e. lack of access to technological devices and internet services). These challenges were further exacerbated by the advent of the pandemic. Academics in this study reported a sense of helplessness associated with being unable to reach students. A study by one author [7] also revealed how academics’ levels of stress increased when the changes to online learning were implemented.

The results of the study highlighted the psychological impact of the COVID-19 pandemic by demonstrating that academics experienced emotional isolation because of the lockdown restrictions and having to adapt to the new working environment. Further to this, the lack of institutional support in terms of managing the changes (i.e. such as having to navigate technology and learning management system), significantly contributed to the isolation and heightened feelings of panic and anxiety. This is confirmed by a study conducted by [7]. Many academics reported feeling ill-prepared and anxious about the adaptation. Additionally, the increased workload was highlighted as significantly contributing to their levels of stress. Another author [4] confirmed this and added that academics reported that their stress was compounded by the fact that they had to teach themselves the new technologies.

The fears of the academics were not restricted to the new working environment; it transcended into their personal lives as well. The findings of this study are supported by another study that found academics to be quite fearful of their health as well as that of their loved ones [8]. This worry added to their fears and anxieties around navigating their new work/life environment.

Some participants in this study seemed to experience a sense of avoidance in relation to their new work/life environment. This is in line with other findings conducted that also reported on participants who tended to use avoidance as a means of coping with the stressors of the pandemic and resulting lockdown restrictions [4, 8]. It must be noted here that their study reported on gender differences as they found this experience to be particular for females, however, this study does not report on gender differences in this regard.

A significant finding of this study was the increase in emotional fatigue reported by most academics. The lack of demarcation between 2020 and 2021 resulted in academics feeling hopeless and helpless in trying to navigate work and life and fulfill all their academic responsibilities. Institutional denial significantly added to this burden and many academics reported experiencing psychological strain because of this. Academics felt that the lack of willingness from institutions to reflect on the impact of the changes which were implemented at the onset of the pandemic directly impacted their ability to pause, take stock, recreate, and innovate. Even though a few academics reported that they tried to be proactive by putting systems in place to support students, they ended up emotionally fatigued. Similar results were reported in a study where their participants experienced emotional fatigue because of the increased workload brought about by additional demands from colleagues and students [4].

## Conclusion

The COVID-19 pandemic and ensuing lockdown restrictions in 2020 and 2021 had a negative psychological impact on many academics in public and private institutions of higher education in South Africa. Institutions must use the lessons learned from 2020 to 2021 to respond to the needs of academics in years to come.

### Limitations and future research

The study naturally had some limitations. The first limitation was the limited participation of male academics. Taking into consideration the interplay between gender, gender roles, mental health, coping mechanisms, and the role of gender in academia, it is recommended that future studies on this topic include equal representation of males and females to gain more insight into the experiences and coping mechanisms employed by male academics.

Another limitation is the small sample size of participants and higher education institutions in the study. A small sample size such as the one in this study potentially impacts the transferability of the findings to other academics at other institutions. It may be that academics in other institutions did not share the same experiences as their contexts may have been different from the ones in this study.

Since this was a cross-sectional research study, future research could be conducted longitudinally. It would be interesting to explore the experiences of academics potentially after six to twelve months after this study to ascertain if they are still experiencing any negative psychological impact of COVID-19, but more importantly, if participants are still employing the coping mechanism they utilised during the pandemic.

### Implications for practice

Given that academics experience constant pressures to adapt to an ever-changing higher education landscape, engage with students, impart knowledge and skills, produce high-quality research and participate in scholarship activities regularly, it is important that institutions ensure that academics are equipped to deal with rapid change in future—assistance that will help ease the transition from one status quo to another—particularly in times of national and/or international turmoil and upheaval.

Institutions must strive to respond to the psychological and emotional needs of academic staff. Institutions must create working environments and foster cultures where the mental well-being of academics is encouraged and even protected. Institutional management and human resource departments at academic institutions can create support groups for academics where they can share common challenges, share examples of new teaching and learning methodologies, as well as organise wellness activities and events to support academics to overcome or reduce some of the negative psychological impact created by the COVID-19 pandemic.

Institutions must use the lessons learned from 2020 to 2021 to respond to the needs of academics in years to come. They must ensure that academics are equipped to deal with rapid change in the future—assistance that will help ease the transition from one status quo to another—particularly in times of national and/or international turmoil and upheaval.

Institutions must strive to respond to the psychological and emotional needs of academic staff (lecturers and researchers). Institutions must create working environments and foster cultures where the mental well-being of academics is encouraged and even protected. Institutional management and human resource departments at academic institutions can create support groups for academics where they can share common challenges, share examples of new teaching and learning methodologies, as well as organise wellness activities and events to support academics to overcome or reduce some of the negative psychological impact created by the COVID-19 pandemic.

## Data Availability

The data analysed during the current study are available from the corresponding author on reasonable request.

## Abbreviations

WHO: World Health Organisation
IIE: The Independent Institute of Education
